# A focus on applying ^63/65^Cu solid-state NMR spectroscopy to characterize Cu MOFs

**DOI:** 10.1039/d4sc90069c

**Published:** 2024-04-16

**Authors:** Zhenfeng Pang, Kong Ooi Tan

**Affiliations:** a Laboratoire des Biomolécules, LBM, Département de Chimie, École Normale Supérieure, PSL University, Sorbonne Université, CNRS 75005 Paris France kong-ooi.tan@ens.psl.eu

## Abstract

Metal–organic frameworks (MOFs) are a class of hybrid organic and inorganic porous materials that have shown prospects in applications ranging from gas storage, separation, catalysis, *etc.* Although they can be studied using various characterization techniques, these methods often do not provide local structural details that help explain their functionality. Zhang *et al.* (W. Zhang, B. E. G. Lucier, V. V. Terskikh, S. Chen and Y. Huang, *Chem. Sci.*, 2024, https://doi.org/10.1039/D4SC00782D) have recently exploited ^63/65^Cu solid-state NMR spectroscopy (for the first time) and DFT calculations to elucidate the structures of Cu(i) centers in MOFs. While there are still many challenges in overcoming issues in resolution and sensitivity, this work lays the foundation for further development of solid-state NMR technology in characterizing copper in MOFs or other amorphous solids.

## Abstract 2

Combined solid-state ^63/65^Cu NMR spectroscopy and DFT calculations are powerful tools that can shed light on the local geometries and bonding of different Cu(i) sites in metal–organic frameworks (MOFs) (Zhang *et al.*, *Chem. Sci.*, 2024, https://doi.org/10.1039/D4SC00782D).

## Graphical abstract

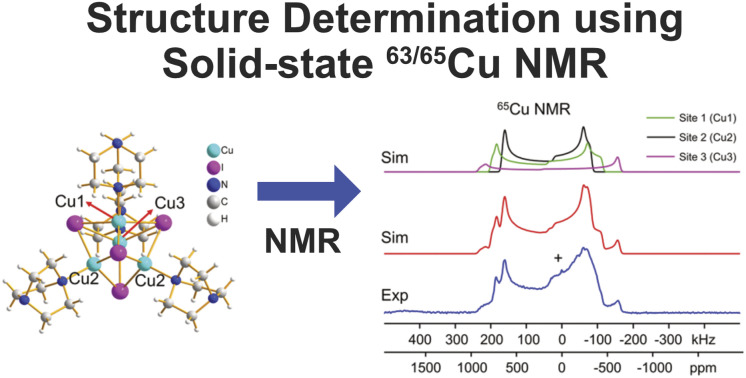

Metal–organic frameworks (MOFs) are a novel class of materials that have gained popularity in recent decades due to their highly porous structures and exceptional tunability, making them attractive candidates for diverse applications such as gas storage, separation, and catalysis.^1^ A critical factor influencing their functional properties is the incorporation of metal centers within the MOF structure. Because of the unique properties of Cu^+^ or Cu^2+^, copper-based MOFs have shown uniquely high photocatalytic activity,^2^ enhanced luminescence properties,^3^ and promising biomedical applications.^4^ Copper can adopt various coordination environments within an MOF, such as two-coordinate linear, three-coordinate trigonal planar, or four-coordinate tetrahedral. The coordination number plays a crucial role in determining the electrical conductivity, structural stability, and reactivity of the MOF. Characterization of these copper centers is challenging, especially for Cu(i), because, unlike Cu(ii), Cu(i) is ‘invisible’ to EPR or UV-vis spectroscopy. Moreover, other spectroscopic techniques, such as powder X-ray diffraction, energy dispersive spectroscopy (EDS), *etc.*, often do not yield high-resolution information on local structural details of the metal centers. Hence, Zhang *et al.* have exploited ^63/65^Cu solid-state nuclear magnetic resonance (NMR) spectroscopy, a powerful method of choice to extract site-specific information of these copper environments in MOFs [(https://doi.org/10.1039/D4SC00782D)^5^ and ref. 6].

Despite the promising aspects, ^63/65^Cu NMR is not commonly employed, mainly because ^63^Cu and ^65^Cu are both spin-3/2 particles that possess quadrupolar interactions that are often too large to be averaged by the magic-angle spinning (MAS) technique.^7^ Hence, their NMR spectra are very broad (>50 kHz) and the poor resolution usually limits the application of ^63/65^Cu NMR to materials with a single site. Although it can be applied to materials with multiple well-defined sites, the results are highly dependent on the quality of spectra fitting, and the conclusions are sometimes subjective or debatable. Moreover, the broad NMR spectra also inherently result in poor NMR sensitivity, which also limits its use to mostly simple 1D NMR experiments. Nevertheless, the linewidths of the ^63/65^Cu NMR spectra performed under static (non-spinning) conditions are primarily determined by chemical-shift anisotropy (CSA) and electric-field gradient (EFG) tensors, which contain rich structural information. Zhang *et al.* have meticulously performed high-field (21.1 T) NMR experiments and density functional theory (DFT) calculations to extract the CSA and EFG tensors of 13 different Cu MOFs. For instance, they showed that the experimental ^63/65^Cu NMR spectra of [Cu_4_I_4_(DABCO)_2_] (Fig. 1) can be very well simulated using the fitted NMR interactions. Moreover, it was shown that the three different Cu sites in the MOF can be remarkably well distinguished, which is a non-trivial task. The experimental NMR data were effectively combined with DFT calculations, so that specific NMR parameters could be confidently assigned to specific copper sites within the MOF structure. This synergy between experiment and theory provides a powerful approach for a comprehensive understanding of the copper environment.

**Fig. 1:**
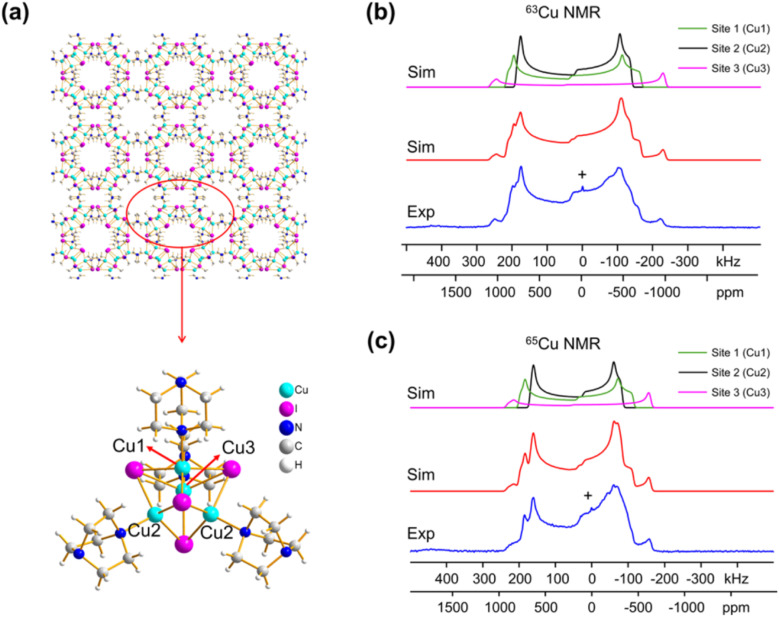
(a) A schematic diagram of [Cu_4_I_4_(DABCO)_2_] featuring three different Cu sites. The experimental (b) ^63^Cu and (c) ^65^Cu static NMR spectra (blue), cumulative simulations (red), and individual Cu site simulations (black, purple, green).

Moreover, Zhang *et al.* also provided a general tool (Fig. 2a) for estimating the chemical environments of Cu(i) *via* their quadrupolar coupling constants (*C*_Q_). This allows them to elucidate the structural change in a Cu MOF participating in an anion exchange reaction. Fig. 2b shows that the *C*_Q_ in the Cu_4_I_4_(DABCO)_2_ MOF has increased significantly when the MOF is soaked in NaNO_3_ or NaClO_4_ solutions. Using the results obtained earlier (Fig. 2a), it was inferred that the Cu(i) center has transitioned from a distorted tetrahedral configuration to either a two- or three-coordinate structure. The results were then compared with PXRD measurements performed on independently synthesized samples, and it was concluded that the connectivities are similar but not identical.

**Fig. 2:**
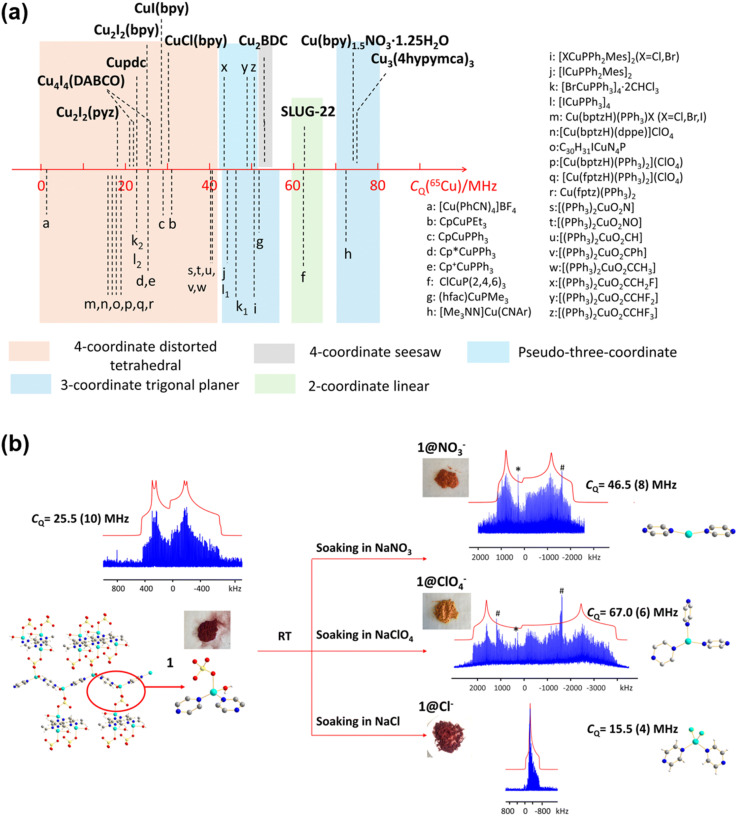
(a) The relationship between the chemical environments of Cu(i) and their *C*_Q_ values. (b) The obvious changes in linewidth and *C*_Q_ indicate a possible structural change of (Cu_2_(SO_4_)(pyz)_2_(H_2_O)_2_) upon addition of various aqueous solutions.

Although the application of solid-state NMR in characterizing ultra-wideline (UW) nuclei still faces challenges due to poor NMR sensitivity and resolution, we are optimistic that new NMR technologies, *i.e.*, ultra-high-field NMR and hyperpolarization, will help circumvent these issues. It is known that ultra-high-field NMR is exceptionally beneficial in characterizing half-integer quadrupolar nuclei (*e.g.*, ^63/65^Cu, ^47/49^Ti, ^95^Mo, ^91^Zr, ^33^S, ^67^Zn, *etc.*) because it grants higher-resolution spectra, in addition to higher sensitivity due to the larger Boltzmann population. The latter feature is due to the fact that the linewidth of the NMR central transitions is inversely proportional to the *B*_0_ magnetic field.^7^ These advantages have recently been exploited to study ^95^Mo, ^33^S, and ^67^Zn using either a commercially available 28.2 T magnet or the world’s highest-field 35.2 T magnet available in the US national facility (MagLab).^8–10^ On the other hand, dynamic nuclear polarization (DNP) is an NMR sensitivity enhancement technique that can usually boost the NMR signals by several orders of magnitude.^11–13^

In conclusion, Zhang *et al.* have shown that ^63/65^Cu NMR spectroscopy is a powerful tool for probing the copper environments within MOFs. By offering site-specific information about the coordination number and geometry of copper centers, it provides crucial insights into the factors governing the properties of MOFs. While challenges remain in overcoming signal broadening, sensitivity limitations, and the need for strategic isotopic enrichment, ongoing advancements in NMR technology, data-processing methods, and integration with other techniques hold immense promise for pushing the boundaries of ^63/65^Cu NMR spectroscopy and further enhancing our understanding of copper-based MOFs. This comprehensive understanding will ultimately pave the way for the rational design of MOFs with tailored properties for specific applications. Moreover, this NMR method could be extended to many different fields involving Cu(i) species, such as catalysis, surface chemistry, solar cells, and biochemistry.

## Author contributions

Z. P. and K. O. T. wrote the manuscript.

## Conflicts of interest

There are no conflicts to declare.

## Supplementary Material
